# Investigating local and long-range neuronal network dynamics by simultaneous optogenetics, reverse microdialysis and silicon probe recordings *in vivo*

**DOI:** 10.1016/j.jneumeth.2014.06.031

**Published:** 2014-09-30

**Authors:** Hannah Taylor, Joscha T. Schmiedt, Nihan Çarçak, Filiz Onat, Giuseppe Di Giovanni, Régis Lambert, Nathalie Leresche, Vincenzo Crunelli, Francois David

**Affiliations:** a Neuroscience Division, School of Biosciences, Cardiff University, Museum Avenue, Cardiff CF10 3AX, UK; b Department of Pharmacology, Faculty of Pharmacy, Instanbul University, Istanbul, Turkey; c Department of Pharmacology and Clinical Pharmacology, Faculty of Medicine, Marmara University, Istanbul, Turkey; d Department of Biochemistry and Physiology, University of Malta, Malta; e Sorbonne Universités, UPMC Univ Paris 06, UM CR18, Neuroscience Paris Seine (NPS), Paris F-75005, France; f CNRS, UMR 8246, NPS, Paris F-75005, France

**Keywords:** T-type Ca^2+^ channels, HCN channels, Metabotropic glutamate receptors, EEG, Slow waves, Sleep spindles, Delta waves, Thalamus, Cortex

## Abstract

**Background:**

The advent of optogenetics has given neuroscientists the opportunity to excite or inhibit neuronal population activity with high temporal resolution and cellular selectivity. Thus, when combined with recordings of neuronal ensemble activity in freely moving animals optogenetics can provide an unprecedented snapshot of the contribution of neuronal assemblies to (patho)physiological conditions *in vivo*. Still, the combination of optogenetic and silicone probe (or tetrode) recordings does not allow investigation of the role played by voltage- and transmitter-gated channels of the opsin-transfected neurons and/or other adjacent neurons in controlling neuronal activity.

**New method and results:**

We demonstrate that optogenetics and silicone probe recordings can be combined with intracerebral reverse microdialysis for the long-term delivery of neuroactive drugs around the optic fiber and silicone probe. In particular, we show the effect of antagonists of T-type Ca^2+^ channels, hyperpolarization-activated cyclic nucleotide-gated channels and metabotropic glutamate receptors on silicone probe-recorded activity of the local opsin-transfected neurons in the ventrobasal thalamus, and demonstrate the changes that the block of these thalamic channels/receptors brings about in the network dynamics of distant somatotopic cortical neuronal ensembles.

**Comparison with existing methods:**

This is the first demonstration of successfully combining optogenetics and neuronal ensemble recordings with reverse microdialysis. This combination of techniques overcomes some of the disadvantages that are associated with the use of intracerebral injection of a drug-containing solution at the site of laser activation.

**Conclusions:**

The combination of reverse microdialysis, silicone probe recordings and optogenetics can unravel the short and long-term effects of specific transmitter- and voltage-gated channels on laser-modulated firing at the site of optogenetic stimulation and the actions that these manipulations exert on distant neuronal populations.

## Highlights

• Optogenetics and microdialysis can be successfully combined.
• How to manipulate circuits of spontaneous and evoked activities with drugs and lights?
• Thalamic control of delta waves and sleep spindles.

## 1 Introduction

Excitation and inhibition of selected neuronal populations provide insights into their role in physiological functions and pathological conditions as well as the effects that these localized manipulations have on distant neuronal assemblies. Among the various available *in vivo* techniques (e.g. electrical stimulation, electrolytic and chemical lesions, cooling, transcranial magnetic stimulation, pharmacological activation or block of membrane channels), optogenetics with its cell-specific expression and millisecond time-scale activation of light-sensitive proteins has proved to be a major technical breakthrough (Zhang et al., 2007, Fenno et al., 2011). Simultaneous recording of the multi- or single-unit activity of the opsin-transfected neuronal population is routinely used in order to monitor the effectiveness of optical stimulation at the site of light delivery. To this end, optic fibers connected to different kinds of recording electrodes, *i.e.* tetrodes (“optetrodes”) (Anikeeva et al., 2012), silicone probes (“optrodes”) (Kravitz et al., 2010, Royer et al., 2010) or other types (Klorig and Godwin, 2014), have been successfully developed.

Notwithstanding these technical advances, however, the need remains to understand how the voltage- and transmitter-gated channels of the opsin-containing neurons contribute to any given (patho)physiological condition. To address this issue, a recent study has used an optic fiber attached to a metal electrode and a glass capillary for the delivery of a solution containing a selective GABA_A_ receptor antagonist (*i.e.* bicuculline methiodide) to the opsin-transfected population (Berglind et al., 2014). Though successful, this route of drug delivery suffers from a number of potential drawbacks, including (i) mechanical instability of the neuronal tissue at the time of injection (with chances of loosing the recorded neurons and thus eliminating the possibility of recording the same neurons before and during drug application), (ii) delivery of an unknown drug concentration at, and around, the site of injection, and (iii) poor control of the spatial extent of drug action. In contrast, reverse microdialysis is known (Höcht et al., 2007, Chan and Chan, 1999) to provide (i) mechanical stability of the neural tissue during drug delivery (enabling the experimenter to monitor drug effects on the same neurons before, during and after drug injection), (ii) measurement of the drug concentration at the site of delivery (by collecting the efflux from the microdialysis probe outlet tube), (iii) a steady-state drug concentration suitable for investigating changes in single neuron and neuronal population activities during prolonged application (*i.e.* hours and possibly days) and (iv) the possibility of monitoring local brain tissue change in neurotransmitter and neuromodulators induced by the drug (by collecting the efflux from the microdialysis probe outlet tube) (Westerink and De Vries, 2001).

Here, we describe the use of reverse microdialysis for drug delivery at the site of channelrhodopsin-2 (ChR2) activation while simultaneous recording with a silicone probe the activity of single neurons during optogenetic activation *in vivo*. We used blockers of two voltage-dependent channels, *i.e.* TTA-P2, a T-type Ca^2+^ channels (T-channels) antagonist (Shipe et al., 2008, Uebele et al., 2009, Dreyfus et al., 2010) and ZD7288 (an hyperpolarization-activated, cyclic nucleotide gated-channel, HCN, antagonist) (BoSmith et al., 1993, Harris and Constanti, 1995, Williams et al., 1997, Hughes et al., 1998), and a ligand-gated channel, *i.e.* LY367385 (a metabotrobic glutamate receptor 1a (mGluRs) antagonist) (Clark et al., 1997, Hughes et al., 2002). As proof of principle, we present experiments on optogenetic excitation of ChR2-transfected thalamocortical (TC) neurons in the thalamic ventrobasal (VB) complex combined with single unit recordings and microdialysis in the same nucleus, and EEG recordings in the somatotopically connected primary somatosensory cortex in anesthetized and freely moving rats.

## 2 Materials and methods

All experimental procedures were carried out in accordance with the UK Animals (Scientific Procedure) Act, 1986, and local ethics committee guidelines. All efforts were made to minimize animal suffering and the number of animals used. Experiments were performed on adult male Wistar rats (260–400 g, Harlan Laboratories, UK) which were maintained on a normal diet and under an 8.00am–8.00pm light-on regime.

### 2.1 Experiments in anesthetized rats

**Fig. 1:**
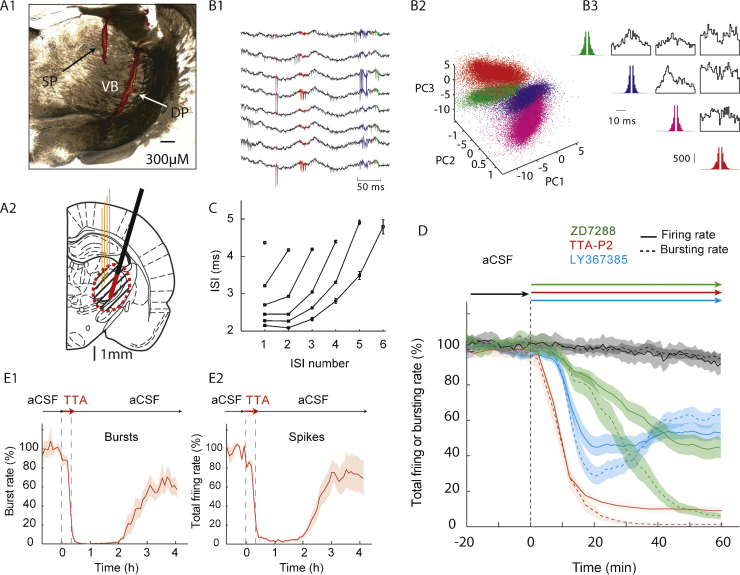
Using a silicone probe to characterize the time-course and diffusion area of action for drugs applied by reverse microdialysis in anesthetized rats. (A1) Coronal brain section showing the position of a microdialysis probe (DP) (inserted with a 16° angle with respect to the vertical axis), and a silicone probe (SP) in the VB (the tracks of both probes are stained red by the fluorescent dye, see Section 2). (A2) Schematic drawing (AP = −3.14 from bregma, Paxinos and Watson, 2007) showing the position of the four shanks of the silicone probe (orange) and microdialysis probe (black) with its active membrane shown in red. The dashed area indicates the zone of diffusion of the drug around the dialysis probe (see text for additional details). (B1) Eight-channel raw traces recording. Spikes from 4 neurons are colored according to the output of the clustering shown in (B2). The 4 clusters are represented in a 3-principal-component (PC) space. (B3) Autocorrelograms (in color) and crosscorrelograms (black) of 4 isolated units recorded simultaneously. (C) Plot showing the characteristic signature of T-type Ca^2+^ channel-mediated bursts of VB TC neurons (ISI: inter-spike interval). (D) Effect on the burst rate (dash line) and firing rate (continuous line) of reverse microdialysis-applied 500 μM ZD7288 (green), 300 μM TTA-P2 (red) and 5 mM LY367385 (blue) recorded within 500 μm from the microdialysis probe. The start of drug dialysis is at time 0. (E) The effect of 300 μM TTA-P2 on burst (E1) and total firing (E2) is reversible. In D and E, shaded areas indicate ±SEM.
(4) a 32-channel silicone probe (10 mm length, 4-shanks interspaced by 200 μm) (Buzsaki32L-CM32, NeuroNexus Technologies) was slowly lowered until it reached the most dorsal portion of the VB (DV = −4.5 mm). During the experiment the probe was then moved while searching for units until the bottom of the VB (DP = −6.5 mm) (Fig. 1A1 and A2).

Anesthesia was induced with 5% isoflurane, followed by an intraperitoneal (ip) injection of ketamine (120 mg/kg) and xylazine (20 mg/kg). Anesthesia was then maintained by constant delivery of ketamine (42 mg/kg/h) and xylazine (7 mg/kg/h) *via* an ip catheter connected to a pump (NewEra NE-300 syringe pump). Body temperature was maintained at 37 °C with a heating pad and rectal probe. The following procedures were carried out:

(1) epidural gold-plated EEG screws (Svenska Dentorama, POS-330, G-P screw posts con.S1) were placed in holes drilled in the skull over the frontal (AP = +2 mm, ML = ±2 mm) and parietal cortices (AP = −2 mm, ML = ±5.5 mm) (these and all other coordinates are relative to bregma) (Paxinos and Watson, 2007);
(2) a 1 mm-diameter hole was drilled unilaterally above the VB (ML = +2.8 mm, AP = −3.2 mm) and the dura was carefully removed with the tip of a small needle under microscope control (this hole was later used for inserting the silicone probe, see step (4) below);
(3) through another 1 mm-diameter hole drilled lateral to the first hole a microdialysis probe (CMA 12 Elite, 2 mm dialysis membrane length, 20 kDa cutoff, well above the molecular weight of the drug used in this study TTA-P2 (431 Da), Zd7288 (292.81 Da), LY367385 (209.20 Da)) was slowly (500 μm every 5 min) lowered at a 16° angle with respect to the vertical axis (Fig. 1A1 and A2), such that its final position rested between 0.05 and 1 mm away from the calculated position of the tip of the silicone probe to be inserted. The microdialysis pump delivered a constant flow rate of 1 μL/min of artificial cerebrospinal fluid (aCSF) bought from Tocris (ref. 3525) which contains the following Final ion concentrations (in mM): Na 150; K 3.0; Ca 1.4; Mg 0.8; P 1.0; Cl 155. 4% DMSO was added for TTA-P2 dissolution;

At the end of the experiments, the rats were killed with an overdose of urethane (40%). The microdialysis probes were placed in eppendorf tubes with distilled water and cleaned by pushing distilled water with the pump with the same flow rate as for experiments during couple of hours.

### 2.2 Experiments in freely moving rats

**Fig. 2:**
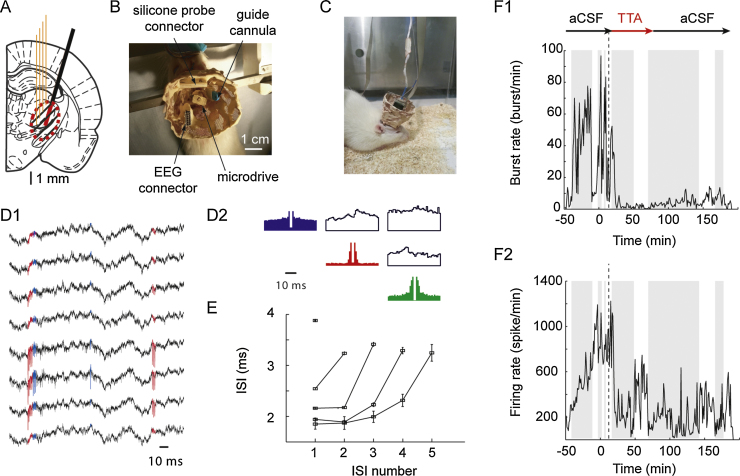
Combined reverse microdialysis and silicone probe recordings in freely moving rats. (A) Schematic drawing (AP = −3.14 from bregma, Paxinos and Watson, 2007) showing the position of the four shanks of the silicone probe (orange) and the microdialysis probe (black) with its active membrane shown in red. The dashed area indicates the zone of diffusion of the drug around the dialysis probe (see text for additional details). (B) Top view of the implant while the rat is positioned in the stereotaxic frame under isoflurane anesthesia, just before the insertion of the microdialysis probe. (C) Picture of the full implant in a freely moving rat. (D) Raw traces from 8 channels of the silicone probe (D1) obtained from a freely moving rat implanted as shown in C. Autocorrelograms (color) and crosscorrelograms (black) of 3 isolated neurons are shown in D2. (E) Plot showing the characteristic signature of T-type Ca^2+^ channel-mediated bursts recorded from a VB TC neuron in a freely moving rat. (F) Effect of reverse microdialysis application of 300 μM TTA-P2 on burst (F1) and total firing (F2) rate of the 3 neurons isolated as in D1-D2. Shaded and white areas indicate period of non-REM sleep and wakefulness, respectively.
(4) the microdrive-mounted silicone probe was slowly lowered stereotaxically to a final position of DV = −4.5 mm, corresponding to the most ventral portion of the VB;
(5) the craniotomy was sealed with a mixture of Mineral Oil (NF/FCC, O121-1, Fisher Scientific) paraffin oil and paraffin granules (P31-500, Fisher Scientific);
(6) EEG electrodes were implanted over the frontal and parietal cortices, as described above, and secured with Metabond and grip cement;
(7) a miniature Faraday-cage made of copper mesh and grip cement (as described in Vandecasteele et al., 2012) was then prepared and fixed to the skull with grip cement (Fig. 2B and C).

For chronic implantation surgery, rats were anesthetized with isoflurane (5%) followed by an ip injection of ketamine (120 mg/kg) and xylazine (20 mg/kg). Additional injections of anesthetics were given when necessary. A 32-channel, 4-shank silicone probe (as above, but of an H-type configuration, Buzsaki32L-H32, 21 mm, NeuroNexus Technologies) was connected to custom-built microdrive, as described in Vandecasteele et al. (2012). The H-type configuration was chosen because it contains an additional flexible conductive ribbon inserted between the omnetics connector and the shanks of the electrode, which allows complete freedom for positioning the electrode shanks on the moveable part of a microdrive. The following procedures were carried out:

(1) an EMG electrode was implanted in the neck muscle;
(2) a small craniotomy was performed above the zone of interest (*i.e.* the VB) for the silicone probe insertion (AP = −3.2 mm, ML = +2.8 mm, about 1.5 mm of diameter).
(3) another hole 1.8 mm caudal (or lateral) was drilled and a microdialysis guide cannula was implanted in the same AP (or ML) plane at DV = −4.5 mm depth and at an angle of 16° to the vertical axis, and then fixed to the skull with Metabond and grip cement (Fig. 2A and B);

When microdialysis probes were implanted bilaterally in the VB without a silicone probe, the guide cannulae were implanted vertically in the VB using the above-mentioned stereotaxic co-ordinates and procedures. Rats were allowed to recover from the implantation surgery for at least 5 days before experiments commenced.

### 2.3 Assembling silicone probe and optic fiber

We used the H-type silicone probe (Buzsaki32L-H32_21 mm, Neuronexus) since it allowed freedom of easily positioning its shanks along the optic fiber (CFM12L20, 200-μm multimode, 0.39 NA, 12 mm long optic fiber, Thorlabs). The silicone probe was attached to the optic fiber using the following steps:

(1) a thin (∼100 μm diameter) wire was first glued (with Loctite Super glue, Henkel) to the epoxy-covered section of the silicone probe which is located between the ribbon and the shanks. This wire was then used for handling the shanks;
(2) once the glue was fully dried, the wire was placed inside a croc third hand which was flattened and taped at its contact sites to secure the handling of the wire. The ribbon-attached connector was taped to a crocodile clip (mounted on a magnetic stand) to avoid movements during handling;
(3) the crocodile clip was rotated in a way that the 4 shanks of the silicone probe were positioned in the horizontal plane;
(4) the optic fiber was positioned horizontally at the same level using a separate clamp;
(5) the silicone probe and optic fiber were superimposed and aligned under a microscope using a digital caliper so that the tip of the silicone probe shanks were protruding about 500 μm from the end of the optic fiber. In order to minimize direct light artifacts (see http://www.openoptogenetics.org/index.php?title=Light-Induced_Artifact), the recording sites of the silicone probe were facing away from the optic fiber;
(6) small drops of glue were added half way along the silicone probe shanks and close to the tips to secure the optic fiber;
(7) once the assembly of the silicone probe and fiber optic (*i.e.* the optrode) was dry, the handling wire was trimmed as short as possible with sharp cutting pliers;
(8) the electrode connector was cemented with grip cement to the optic fiber metallic ferrule which was scuffed to ensure adhesion. An additional small plastic rod was glued to the optrode in order to facilitate its positioning in the clamp that we used for the microdialysis guide cannula manipulation before insertion.

After soaking overnight in distilled water and ethanol, the optrode could be used in multiple animals (other cleaning solutions can be found on the NeuroNexus website).

### 2.4 Viral injection

pAAV-CaMKII_-hChR2(H134R)-mCherry plasmids (K.Deisseroth laboratory, Addgene plasmid 26975) packaged into recombinant AAV2 vectors and serotyped with AAV1 coat proteins, were titered to 1.14 × 10^13^ genome copies/ml (GC) (University of Pennsylvania Vector Core). Concentrated stock virus was diluted with 0.1MPBS tinted with Fast Green FCF (Sigma), giving a final viral concentration of 5.70 × 10^8^ to 2.28 × 10^9^ GC/μl for injection. The viral injection procedure was carried out as previously described (David et al., 2013).

### 2.5 Experiments with silicone probe, microdialysis probe and optic fiber

The following procedures were carried out in rats that had received viral injection:

(1) under ketamine/xylazine anesthesia, doses and route as described above for experiments performed under anesthesia, two holes were drilled: one for the optrode (AP = −3.2 mm, ML = −2.8 mm), and one for the microdialysis probe (AP = −3.2 mm, ML = −4.6 mm);
(2) the microdialysis probe was slowly inserted with a 16° angle toward the site of the silicone probe until its tip rested into a final position corresponding to DV = −6.5 mm, and left it attached to the stereotaxic arm;
(3) finally, the optrode was connected to a compatible patch-cord and 473 nm laser diode (70 mW Stradus; Vortran Laser Technology Inc., California, USA), and stereotaxically lowered vertically until the tip of the silicone probe reached the most dorsal section of the VB.

Note that for experiments in freely-moving animals, the same sets of procedures can be followed except that a guide cannula, instead of the microdialysis probe, is inserted during step (2) above. The dummy probe is then removed before the experiments and replaced by a microdialysis probe. Moreover, for experiments in freely moving rats the optrode can be mounted on a microdrive.

### 2.6 Optical stimulation and recording

The silicone probe and EEG wires were connected to a Digital Lynx 10SX recording system (with Hybrid Input Boards; Neuralynx) *via* ADPT-HS36-N2T-32 and HS-36 unity gain preamplifiers. Electrical signals and the timing of the light stimulation events were simultaneously recorded using Cheetah 5 Data Acquisition software (Neuralynx), while digital laser modulation was controlled with pClamp software and a 1322A Digidata (Molecular Devices), synchronized with the Digital Lynx 10SX. Laser output power was adjusted as required and quantified with a digital power meter and photodiode sensor (PM120D; Thorlabs) (for additional details, see David et al., 2013).

### 2.7 Data acquisition

In experiments where no optogenetics was used, TC neuron signals were amplified with an Omnetics preamplifier (gain 20, bandwidth 0.8 Hz to 54 kHz) or a Plexon recorder/64 channel amplifier (gain 7500–12,500, bandwidth 1–6000 Hz, Plexon). The EEG signal was amplified with a combination of SuperTech Bio-AMP (Pecs) pre- (bandwidth 0.1–500 Hz) and main-amplifiers (bandwidth DC to 500 Hz). When combined unit and EEG recordings were made, signals were digitized with a Plexon recorder/64 system at 20 kHz with 16-bit resolution. EEG recordings were digitized using the Plexon recorder input *via* the IP16 event input breakout panel. The digitized data were converted to Spike2 format (version 5.13, CED). For all further analyses, data were converted to a raw binary format using tools of the freely available Klusters, Neuroscope, and NDManager software suite (Hazan et al., 2006). EEG data were low-pass filtered with a windowed sinc filter at 100 Hz and downsampled to 200 Hz.

### 2.8 Data analysis

Spike sorting and data preprocessing were performed with the Klusters, Neuroscope, NDManager and Klustakwik software suites (Harris et al., 2000, Hazan et al., 2006). All other analyses were performed with routines based on the free toolboxes SciPy 0.8 (Jones et al., 2001), OpenElectrophy 0.2 (Garcia and Fourcaud-Trocmé, 2009), running under Python 2.6.6 and MATLAB (R2010b, MathWorks) on a 64-bit Linux computer.

### 2.9 Data processing and spike sorting

**Fig. 3:**
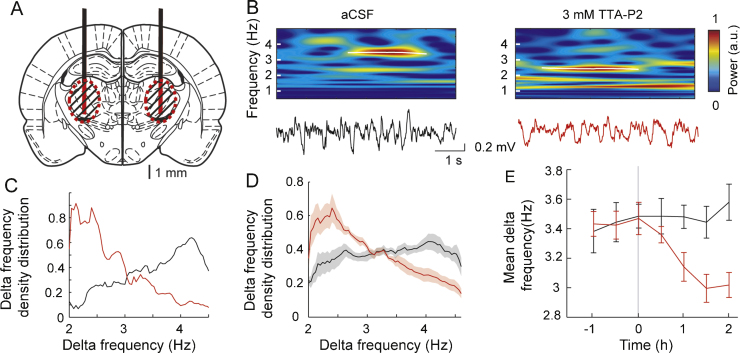
EEG effects of subcortical reverse microdialysis. (A) Schematic drawing (AP = −3.14 from bregma, Paxinos and Watson, 2007) showing the position of the bilaterally implanted microdialysis probes (black) with their active membrane shown in red. The dashed areas indicate the zone of diffusion of the drug around the dialysis probes (see text for additional details). (B) Delta waves detected by wavelet transform (top plots) of the EEG signal (bottom traces) show a clear reduction in frequency during TTA-P2 microdialysis in the VB. (C) Delta frequency distribution in one rat (black: aCSF; red: TTA-P2). (D) Density distribution of delta wave frequency (2–4.5 Hz) (black: aCSF *n* = 13, red: TTA-P2, *n* = 10; shaded areas represent ±SEM). (E) Average (±SEM) delta frequency during aCSF (black) and TTA-P2 (red) reverse microdialysis. The start of the microdialysis application of TTA-P2 is at time 0.

Spike sorting and spindle wave detection was performed as described in David et al. (2013). For delta waves, the EEG signal was convolved with complex Morlet wavelets of 3.0 cycles for sleep delta waves at an interpolated frequency resolution of 0.01 Hz (Kronland-Martinet et al., 1987). Using a wavelet ridge extraction method, each oscillatory epoch of the EEG was extracted with an energy threshold to detect its beginning and end (see Fig. 3B) (Roux et al., 2007, Garcia and Fourcaud-Trocmé, 2009). The boundary frequencies of delta wave detection were chosen as from 2 to 4.5 Hz for delta waves. The threshold was defined as 2 times the average energy during a non-REM sleep period during the control session. Delta waves with less than 4 cycles were discarded. When overlapping oscillations were detected, the wave with the highest energy was selected.

### 2.10 Histology

In order to visualize their position in the area of interest *post hoc*, before insertion into the brain the silicone and microdialysis probes were immersed into an Eppendorf tube containing a dye solution (1% Vybrant DIL cell-labeling solution, Invitrogen V22885 diluted in distilled water and 30% ethanol) for 30 and 5 min, respectively. At the end of the experiments, brains were removed, placed in a 4% PFA solution for 48 h, and then stored in a 0.1 M PBS until required. Sections (100 μm thick) containing the VB were cut with a vibratome (Leica VT1000S) and viewed under a Leica microscope to measure the relative position of the tracks of the microdialysis and silicone probes.

### 2.11 Statistical analysis

Group comparisons were performed using the Mann–Whitney *U* test. All quantitative data in the text and figures are expressed as mean ± SEM.

## 3 Results and discussion

### 3.1 Drug diffusion and time-course

Before combining optogenetics with pharmacological manipulation and neuronal ensemble recording, it is necessary to evaluate the diffusion of the drug in the brain area of interest (the VB in this study) and the time-course of its effects, since these parameters strictly depends on each drug's physico-chemical properties (Westerink and De Vries, 2001). This can be achieved by implanting a silicone probe stereotaxically in the VB to monitor the firing of TC neurons while delivering the drug *via* reverse microdialysis from a neighboring, stereotaxically implanted probe (see Section 2) (Fig. 1A1 and A2). This procedure is better performed under anesthesia since under this condition (i) it is easier to adjust the position of both the silicone and dialysis probes, and (ii) the signal to noise ratio of the electrical recordings is better than that in freely moving animals. Moreover, anesthesia provides a more stable condition to assess the time-course of drug action compared to the rapidly changing vigilance states that occur during recordings in freely moving animals. In this respect, it is also advisable to achieve a stable state of anesthesia by continuous infusion of the anesthetic(s) through a catheter connected to a pump (see Section 2). Thus, following an initial ip injection for the induction of anesthesia, the anesthetic pump was switched on at least 1 h before any electrical recording commences. This procedure ensured a stable baseline of the recorded electrical activity, as indicated by the stability of single cell firing during reverse microdialysis application of aCSF (see black line in Fig. 1D).

Extracellular signals recorded from 1 of the 4 shanks of the silicone probe (Fig. 1B1) showed the characteristic firing pattern of VB TC neurons during anesthesia, *i.e.* a predominance of low threshold, T-type Ca^2+^ channel-mediated bursts (hereafter referred to as T-channel bursts) (Fig. 1C) in well-separated single neurons, as supported by the spike sorting procedures (Fig. 1B2), auto-correlograms and cross-correlograms (Fig. 1B3). In neurons that were located <500 μm from the microdialysis probe, 1 h after the start of the reverse microdialysis of 300 μM the T-channel blocker TTA-P2 both T-channel bursts and total firing (*i.e.* single action potentials plus bursts) were abolished (100 ± 0.2% and 95 ± 2.0% block, respectively) (Fig. 1D, red lines). Moving the silicon probe further away, resulted in a smaller TTA-P2-elicited block (73 ± 8% and 71 ± 7%, respectively) in neurons located between 500 and 800 μm from the microdialysis probe, while at greater distances (*i.e.* >800 μm), no-significant effect of TTA-P2 on T-channel-bursts and total firing was observed (−6 ± 9% and −2 ± 6%, respectively). From this data, the effective “diffusion area” of drug action was estimated, as shown in the shaded area of Fig. 1A2. Larger effective “diffusion areas” are achieved with higher drug concentrations in the inlet tube of the microdialysis probe (not shown). Importantly, the blocking effect of TTA-P2 on T-channel bursts and total firing was not due to mechanical injury to the tissue or rapid formation of gliosis around the microdialysis and silicone probes as it was reversible following a switch back to the aCSF solution (Fig. 1E1 and E2).

As far as the time-course of drug action is concerned, the effect of TTA-P2 started to appear within 5 min from the start of its dialysis into the VB, reaching a maximum and stable block of both bursts and total firing by 30 min, when the dialysis probe was within 500 μm from the silicone probe (green lines in Fig. 1D). Of course, longer delays of drug action were observed for larger relative distances between the two probes (not shown). We also tested the effect of the HCN channel blocker ZD7288 and the mGluR-1a antagonist LY367385, since both of these voltage-dependent and ligand-gated channels are known to be critical for the excitability of TC neurons (Hughes et al., 2002, Hughes et al., 2004, Blethyn et al., 2006). The effect of 500 μM ZD7288 started about 13 min from the start of the dialysis and a maximum and steady block of both bursts and total firing was achieved after 50 min (Fig. 1D, green lines). LY367385 (5 mM) action started 10 min after the beginning of the dialysis with a maximum block of 71 ± 5% and 55 ± 5% of bursts and total firing, respectively, achieved after 20 min (Fig. 1D, blue lines). However, the continuous microdialysis delivery of LY367385 showed that the extent of the block exerted by this antagonist decreased with time, reaching a final steady-state block (bursts: 39 ± 4%, total firing: 49 ± 6%) after 40 min from the start of the dialysis (Fig. 1D, blue lines).

### 3.2 Drug effects on single units in freely moving animals

After establishing the diffusion area, time-course and potency of a drug applied *via* reverse microdialysis in the mechanically and electrically more stable conditions achieved during anesthesia, the same protocol was applied to freely moving animals in order to eliminate any confounding effect of the anesthetic, thus allowing the activity/system of interest to be studied under normal physiological conditions. Because of the role of T-channels in sleep oscillations, single TC neuron firing dynamics during natural sleep were investigated, and for the remainder of this work, only results obtained with TTA-P2 are shown.

During surgery, the microdialysis guide cannula (containing a dummy probe) was stereotaxically implanted first. The silicone probe (carried by the microdrive, see Section 2) was then lowered to approximately 500 μm above the drug diffusion target area (as calculated from the anesthetized experiments) (see Fig. 2A–C). Following 5 days of recovery from surgery, the rats were then habituated to sleep in the recording cage for at least 4 h/day for 4 days. During these habituation sessions (Fig. 2C), the silicone probe was connected to the amplifier and very slowly lowered until all shanks reached the top of the VB (Fig. 2A), as indicated by the predominance in single unit recordings of T-channel bursts during non-REM sleep (Fig. 2D1, D2, and E) and tonic firing during wakefulness.

On the first day of experiments, the rats were lightly anesthetized with isoflurane (2%) and placed into the stereotaxic frame (Fig. 2B). The dummy probe was removed from the guide cannula and the microdialysis probe was manually inserted into the guide cannula without penetrating in the brain. The dialysis probe was slowly stereotaxically lowered (500 μm every 5 min) into the brain. The animal was then released from the stereotaxic frame and allowed to wake up into the recording cage (which generally required about 20 min). The inlet and outlet tubes of the microdialysis probe were connected to the pump and the aCSF solution allow to flow through while neuronal activity was monitored from the silicone probe. At this stage, the silicone probe was lowered using the microdrive until the tips of the shanks were (on the basis of the stereotaxic co-ordinates) just above the middle of the VB (Fig. 2A).

Once well separated units were found on a number of shanks, or on a number of contact points on a shank (Fig. 2D1 and D2), they were recorded for a period of at least 40–60 min while continuing to deliver aCSF *via* the microdialysis probe: this ensured a stable baseline activity during different behavioral conditions (*i.e.* wake and natural sleep) before drug application was commenced. If the single unit activity recorded during this period was not stable, either this control period was extended or the silicone probe was slightly lowered down using the microdrive until new stable single units were encountered.

Following the recording of a stable control period, the inlet tube of the microdialysis probe was switched to a solution containing TTA-P2, while continuing to record from the same units as in the control period during aCSF dialysis. Following off-line spike sorting (Fig. 2D2 and E) (as described for the anesthetized experiments), TTA-P2 could be observed to block T-channel bursts and total firing during natural sleep and in the wake state (Fig. 2F1 and F2). Similar recording could be performed for a few consecutive days by leaving the microdialysis probe in the tissue after an experiment but washing out the drug with aCSF. In our experience, it was better not to replace the dialysis probe, since the damage to the surrounding neural tissue associated with multiple probe insertions may compromise the physiological integrity of the tissue. However, it is of note that gliosis around a probe left *in situ* may reduce the diffusion of a drug over time. If a dialysis probe needs replacing, it is important to move the microdrive upwards, thus lifting the silicone probe away from the microdialysis probe track, since the removal and re-insertion of a dialysis probe can damage the silicone probe.

These results demonstrate the possibility of monitoring the effect of pharmacological manipulations of neuronal ensemble activity in freely moving conditions with a silicone probe and reverse microdialysis, as it was shown for tetrode recordings and reverse microdialysis (Van Duuren et al., 2007).

### 3.3 Drug effect on remote areas: EEG delta waves

Based on the knowledge of the drug effect in the VB from our previous experiments in both anesthetized and freely moving conditions, it then became feasible to investigate how the changes of thalamic activity in the VB might influence neuronal ensemble activity in its main projection area, the primary somatosensory cortex. Thus, a different group of rats were implanted bilaterally with 1 or 2 microdialysis guide cannula(e) in each VB (Fig. 3A) together with frontal and parietal EEG electrodes and an EMG electrode in the neck muscle. Following recovery from surgery, the dummy probes were removed and microdialysis probes were slowly inserted into the guide cannula 24 h before the recording commenced. The EEG was recorded for 4 h: half of the rats were recorded for 2 h of control infusion (*i.e.* aCSF) followed by 2 h of TTA-P2 (300 μM) injection, while the remaining rats were recorded for 4 h of aCSF administration. At the end of the experiment, the microdialysis probes were gently removed and replaced by dummy probes. One week later, the same protocol was repeated, with the animals that had been injected with TTA-P2 receiving now an aCSF microdialysis administration for 4 h, and vice versa.

As shown in Fig. 3B, blocking T-channels in the VB with reverse microdialysis application of 300 μM TTA-P2 was able to alter significantly EEG delta (2–4.5 Hz) waves (detected as transient increases of the power of the wavelet transform). In fact, the density distribution of the frequency of delta waves (Fig. 3C and D) indicated a major change, with the density of the upper band of the delta wave frequency decreasing and that of the lower band increasing. Thus, the average delta frequency was significantly reduced from 3.5 ± 0.1 Hz to 2.9 ± 0.1 Hz after 2 h of TTA-P2 microdialysis compared to aCSF infusion (*p* < 0.01, Mann–Whitney *U* test, TTA-P2 *n* = 10 rats, aCSF: *n* = 13 rats) (Fig. 3E).

These data support the hypothesis (Leresche et al., 1990, Leresche et al., 1991, McCormick and Pape, 1990, Dossi et al., 1992) that thalamic T-channels are involved in the expression of delta waves in the neocortex during natural sleep. The validity of these data is supported by the results of our initial experiments in anesthetized rats, which clearly identified the area of effective drug action around the microdialysis probe and the degree of block achieved by the antagonist. Indeed, this selective pharmacological approach may under certain conditions be more solid than a transgenic approach (Lee et al., 2004, Anderson et al., 2005), in which recombination and/or compensation may occur, and faster than RNA silencing experiments.

### 3.4 Investigating the contribution of a voltage-gated channel population during optogenetic experiments

While optogenetic activation or inhibition of a specific neuronal ensemble can be achieved with high temporal resolution and cellular selectivity (Zhang et al., 2007), this technique does not allow investigation of the role played by the voltage- and transmitter-gated channels of the opsin-transfected neurons. In particular, whereas it has been demonstrated that selective block of thalamic T-channels abolishes sleep spindles of natural sleep (Astori et al., 2011, David et al., 2013), it is interesting to investigate whether the thalamic input to cortex is sufficient to entrain cortical activity at spindle frequency even in the absence of thalamic T-channels. We therefore combined reverse microdialysis application of TTA-P2 in the VB with optogenetic stimulation of ChR2-transfected TC neurons while recording their firing with a silicone probe.

**Fig. 4:**
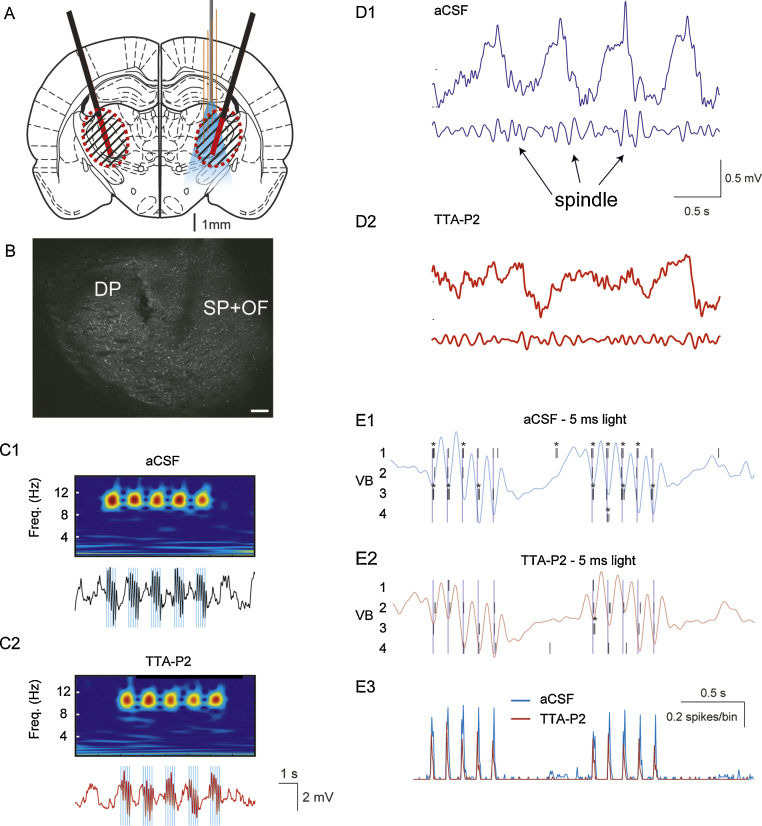
Combined optogenetics, reverse microdialysis and silicone probe recordings. (A) Schematic drawing (AP = −3.14 from bregma, Paxinos and Watson, 2007) showing the position in the VB of the optrode (with the four shanks of the silicone probe in orange and the attached optic fiber in gray) and the microdialysis probes (black) with their active membranes shown in red. (B) Coronal brain section indicating the tracks of the microdialysis probe (DP) and the optrode (SP + OF). (C) Wavelet transform (top) and ipsilateral EEG (bottom) during laser light stimulation in the VB, consisting of 5 trains of 5 (5 ms) pulses at 10 Hz (laser intensity: 40 mW) during reverse microdialysis of aCSF (C1) and TTA-P2 (C2) in an anesthetized rat. Blue lines mark the time of the laser light stimulation. (D) Spindle activity on raw (top) and band-passed signal (low trace) before (D1) and after (D2) TTA-P2 microdialysis. Calibrations in D1 also apply to D2. (E) Raster plots (and superimposed respective EEG traces) showing the activity of 4 VB TC neurons in response to the light pulses during aCSF (E1) and TTA-P2 (E2) microdialysis. Light pulse-triggered spike rates (E3) from 15 stimulation epochs.

Rats which had been injected bilaterally 3 weeks earlier with pAAV-Chr2 were anesthetized and implanted bilaterally with epidural EEG electrodes and microdialysis probes and unilaterally with an optrode (see Section 2) (Fig. 4A and B). While dialyzing aCSF and recording TC neuron activity within the drug diffusion area in the VB, trains of 5 short (*i.e.* 5 ms) light pulses at 10 Hz delivered to TC neurons were able to entrain cortical activity in primary somatosensory cortex (Fig. 4C1). After 40 min from the start of TTA-P2 reverse microdialysis in the VB, spontaneously occurring spindles were abolished in the EEG and thalamic LFP recordings (Fig. 4D1 and D2), but the same light stimulation protocol induced an EEG pattern similar to that observed in the absence of the drug (Fig. 4C2). Analysis of the single TC neuron firing before TTA-P2 application revealed that the vast majority of the light pulses were able to evoke typical T-channel bursts of action potentials (Fig. 4E1, asterisks). Interestingly, whereas most of the spontaneous bursts were blocked by TTA-P2 application (not shown), light pulses were still able to evoke strong firing mostly consisting of single spikes (Fig. 4E2). Indeed, light pulse-triggered average of the TC neuron firing clearly revealed little difference in the light-elicited firing of these cells before and during reverse microdialysis application of TTA-P2, *i.e.* during T-channel block (Fig. 4E3). This observation might explain the recent surprising results of Lee et al. (2013), showing that spindles can be observed during natural sleep in CaV3.1 knockout mice.

## 4 Conclusions

We have demonstrated that the combined use of silicone probe recordings, optogenetics and reverse microdialysis can be successfully used to investigate the contribution of voltage- and transmitter-gated channels to the firing dynamics of localized neuronal populations and their effects on distant neuronal ensembles *in vivo*. This combination of techniques, therefore, provides a solid methodological approach until light-evoked stimulation or inhibition of selected populations of voltage- and transmitter-gated channels become routinely available.

## References

[bib0005] Anderson M.P., Mochizuki T., Xie J., Fischler W., Manger J.P., Talley E.M. (2005). Thalamic Cav3.1 T-type Ca^2+^ channel plays a crucial role in stabilizing sleep. Proc Natl Acad Sci USA.

[bib0010] Anikeeva P., Andalman A.S., Witten I., Warden M., Goshen I., Grosenick L. (2012). Optetrode: a multichannel readout for optogenetic control in freely moving mice. Nat Neurosci.

[bib0015] Astori S., Wimmer R.D., Prosser H.M., Corti C., Corsi M., Liaudet N. (2011). The Ca(V)3.3 calcium channel is the major sleep spindle pacemaker in thalamus. Proc Natl Acad Sci USA.

[bib0020] Berglind F., Ledri M., Sørensen A.T., Nikitidou L., Melis M., Bielefeld P. (2014). Optogenetic inhibition of chemically induced hypersynchronized bursting in mice. Neurobiol Dis.

[bib0025] Blethyn K.L., Hughes S.W., Tóth T.I., Cope D.W., Crunelli V. (2006). Neuronal basis of the slow (<1 Hz) oscillation in neurons of the nucleus reticularis thalami in vitro. J Neurosci.

[bib0030] BoSmith R.E., Briggs I., Sturgess N.C. (1993). Inhibitory actions of ZENECA ZD7288 on whole-cell hyperpolarization activated inward current (If) in guinea-pig dissociated sinoatrial node cells. Br J Pharmacol.

[bib0035] Chan S.H.H., Chan J.Y.H. (1999). Application of reverse microdialysis in the evaluation of neural regulation of cardiovascular functions. Anal Chim Acta.

[bib0040] Clark B., Baker S., Goldsworthy J., Harris J., Kingston A. (1997). (+)-2-Methyl-4-carboxyphenylglycine (LY367385) selectively antagonises metabotropic glutamate mGluR1 receptors. Bioorg Med Chem Lett.

[bib0045] David F., Schmiedt J.T., Taylor H.L., Orban G., Di Giovanni G., Uebele V.N. (2013). Essential thalamic contribution to slow waves of natural sleep. J Neurosci.

[bib0050] Dossi R.C., Nuñez A., Steriade M. (1992). Electrophysiology of a slow (0.5–4 Hz) intrinsic oscillation of cat thalamocortical neurones in vivo. J Physiol.

[bib0055] Dreyfus F.M., Tscherter A., Errington A.C., Renger J.J., Shin H.-S., Uebele V.N. (2010). Selective T-type calcium channel block in thalamic neurons reveals channel redundancy and physiological impact of I(T)window. J Neurosci.

[bib0065] Fenno L., Yizhar O., Deisseroth K. (2011). The development and application of optogenetics. Annu Rev Neurosci.

[bib0070] Garcia S., Fourcaud-Trocmé N. (2009). OpenElectrophy: an electrophysiological data- and analysis-sharing framework. Front Neuroinform.

[bib0075] Harris K.D., Henze D.A., Csicsvari J., Hirase H., Buzsaki G. (2000). Accuracy of tetrode spike separation as determined by simultaneous intracellular and extracellular measurements. J Neurophysiol.

[bib0080] Harris N.C., Constanti A. (1995). Mechanism of block by ZD 7288 of the hyperpolarization-activated inward rectifying current in guinea pig substantia nigra neurons in vitro. J Neurophysiol.

[bib0085] Hazan L., Zugaro M., Buzsáki G. (2006). Klusters, NeuroScope, NDManager: a free software suite for neurophysiological data processing and visualization. J Neurosci Methods.

[bib0090] Höcht C., Opezzo J.A.W., Taira C.A. (2007). Applicability of reverse microdialysis in pharmacological and toxicological studies. J Pharmacol Toxicol Methods.

[bib0095] Hughes S.W., Cope D.W., Blethyn K.L., Crunelli V. (2002). Cellular mechanisms of the slow (<1 Hz) oscillation in thalamocortical neurons in vitro. Neuron.

[bib0100] Hughes S.W., Cope D.W., Crunelli V. (1998). Dynamic clamp study of Ih modulation of burst firing and delta oscillations in thalamocortical neurons in vitro. Neuroscience.

[bib0105] Hughes S.W., Lörincz M., Cope D.W., Blethyn K.L., Kékesi K.A., Parri H.R. (2004). Synchronized oscillations at alpha and theta frequencies in the lateral geniculate nucleus. Neuron.

[bib0110] Jones E., Oliphant T., Peterson P. (2001). http://www.scipy.org/.

[bib0115] Klorig D.C., Godwin D.W. (2014). A magnetic rotary optical fiber connector for optogenetic experiments in freely moving animals. J Neurosci Methods.

[bib0120] Kravitz A.V., Freeze B.S., Parker P.R.L., Kay K., Thwin M.T., Deisseroth K. (2010). Regulation of parkinsonian motor behaviours by optogenetic control of basal ganglia circuitry. Nature.

[bib0125] Kronland-Martinet R., Morlet J., Grossmann A. (1987). Analysis of sound patterns through wavelet transforms. Int J Pattern Recogn Artif Intell.

[bib0130] Lee J., Kim D., Shin H.-S. (2004). Lack of delta waves and sleep disturbances during non-rapid eye movement sleep in mice lacking alpha1G-subunit of T-type calcium channels. Proc Natl Acad Sci USA.

[bib0135] Lee J., Song K., Lee K., Hong J., Lee H., Chae S. (2013). Sleep spindles are generated in the absence of T-type calcium channel-mediated low-threshold burst firing of thalamocortical neurons. Proc Natl Acad Sci USA.

[bib0140] Leresche N., Jassik-Gerschenfeld D., Haby M., Soltesz I., Crunelli V. (1990). Pacemaker-like and other types of spontaneous membrane potential oscillations of thalamocortical cells. Neurosci Lett.

[bib0145] Leresche N., Lightowler S., Soltesz I., Jassik-Gerschenfeld D., Crunelli V. (1991). Low-frequency oscillatory activities intrinsic to rat and cat thalamocortical cells. J Physiol.

[bib0150] McCormick D.A., Pape H.C. (1990). Properties of a hyperpolarization-activated cation current and its role in rhythmic oscillation in thalamic relay neurones. J Physiol.

[bib0155] Paxinos G., Watson C. (2007).

[bib0160] Roux S.G., Cenier T., Garcia S., Litaudon P., Buonviso N. (2007). A wavelet-based method for local phase extraction from a multi-frequency oscillatory signal. J Neurosci Methods.

[bib0165] Royer S., Zemelman B.V., Barbic M., Losonczy A., Buzsáki G., Magee J.C. (2010). Multi-array silicon probes with integrated optical fibers: light-assisted perturbation and recording of local neural circuits in the behaving animal. Eur J Neurosci.

[bib0170] Shipe W.D., Barrow J.C., Yang Z.-Q., Lindsley C.W., Yang F.V., Schlegel K.-A.S. (2008). Design, synthesis, and evaluation of a novel 4-aminomethyl-4-fluoropiperidine as a T-type Ca^2+^ channel antagonist. J Med Chem.

[bib0175] Uebele V.N., Nuss C.E., Fox S.V., Garson S.L., Cristescu R., Doran S.M. (2009). Positive allosteric interaction of structurally diverse T-type calcium channel antagonists. Cell Biochem Biophys.

[bib0180] Vandecasteele M., S M., Royer S., Belluscio M., Berényi A., Diba K. (2012). Large-scale recording of neurons by movable silicon probes in behaving rodents. J Vis Exp.

[bib0060] Van Duuren E., van der Plasse G., van der Blom R., Joosten RNJMA, Mulder A.B., Pennartz C.M.A. (2007). Pharmacological manipulation of neuronal ensemble activity by reverse microdialysis in freely moving rats: a comparative study of the effects of tetrodotoxin, lidocaine, and muscimol. J Pharmacol Exp Ther.

[bib0185] Westerink B.H., De Vries J.B. (2001). A method to evaluate the diffusion rate of drugs from a microdialysis probe through brain tissue. J Neurosci Methods.

[bib0190] Williams S.R., Turner J.P., Hughes S.W., Crunelli V. (1997). On the nature of anomalous rectification in thalamocortical neurones of the cat ventrobasal thalamus in vitro. J Physiol.

[bib0195] Zhang F., Aravanis A.M., Adamantidis A., de Lecea L., Deisseroth K. (2007). Circuit-breakers: optical technologies for probing neural signals and systems. Nat Rev Neurosci.

